# Expansions of tumor-reactive Vdelta1 gamma-delta T cells in newly diagnosed patients with chronic myeloid leukemia

**DOI:** 10.1007/s00262-022-03312-3

**Published:** 2022-11-14

**Authors:** Andrea Knight, Martin Piskacek, Michal Jurajda, Jirina Prochazkova, Zdenek Racil, Daniela Zackova, Jiri Mayer

**Affiliations:** 1 Faculty of Medicine, Department of Pathological Physiology, Masaryk University, Brno, Czech Republic; 2 Department of Internal Medicine, Hematology and Oncology, Masaryk University and Faculty Hospital Brno, Brno, Czech Republic; 3 Institute of Hematology and Blood Transfusion, Prague, Czech Republic

**Keywords:** Gamma-delta T cells, Chronic myeloid leukemia, Tumor immunotherapy, Clonality

## Abstract

Recent studies have underscored the importance of gamma-delta (γδ) T cells in mediating potent MHC-unrestricted cytotoxicity in numerous malignancies. Here, we analyzed Vδ1 and Vδ2 γδ T cell subsets in newly diagnosed chronic myeloid leukemia (CML) patients (*n* = 40) who had initiated tyrosine kinase inhibitor (TKI) therapy including imatinib (*n* = 22), nilotinib (*n* = 14) and dasatinib (*n* = 4). Patient peripheral blood samples were analyzed at diagnosis and monitored prospectively at 3, 6, 12 and 18 months post-TKI. γδ T cells isolated from healthy donors and CML patients were used against K562, LAMA-84 and KYO-1 cell lines and against primary CML cells in cytotoxicity assays. We found large expansions of Vδ1 and Vδ2 T cells in patients at diagnosis compared to age-matched healthy donors (*n* = 40) (*p* < 0.0001). The γδ T cell reconstitution in patients on imatinib and also on nilotinib showed significant reductions of Vδ1 T cell and Vδ2 T cell absolute counts at 3 months compared to diagnosis. Importantly, Vδ1 and Vδ2 T absolute cell counts remained at normal levels from 3 months throughout the follow-up. Next, we observed susceptibility to specific lysis of primary CML tumor cells by Vδ1 T cells from healthy donors. Furthermore, we determined inherent cytotoxic reactivity by autologous patients’ Vδ1 T lymphocytes against primary CML tumor cells. Finally, the TCR clonality profiles showed in CML patients mostly polyclonal repertoires regardless of the TKI. Our results provide further evidence into γδ T cell antileukemia immunity in CML that might be beneficial for long-term disease control and treatment outcome.

**Supplementary Information:**

The online version contains supplementary material available at 10.1007/s00262-022-03312-3.

## Introduction

Chronic myeloid leukemia (CML) is a myeloproliferative disease characterized by the reciprocal *t*(9;22) translocation, the Philadelphia chromosome, leading to the formation of the oncogenic BCR::ABL1 fusion gene with autonomous tyrosine kinase activity [1, 2]. First-line therapies for CML include the tyrosine kinase inhibitors (TKIs) imatinib, dasatinib, nilotinib and bosutinib [3–6].

These agents induce rapid cytogenetic response in the majority of CML patients in chronic phase (CP) dramatically improving the patient survival [7]. However, persisting BCR-ABL transcripts and residual disease has been demonstrated [8] and even in those patients who achieve and maintain a complete molecular response [9]. Earlier data show that TKI discontinuation may be feasible in a proportion of patients with prolonged molecular remission due to restoration of body’s own immune surveillance during therapy [10].

New molecular biomarkers implicated in immune surveillance that impact disease progression and patient prognosis are extensively studied in cancer including CML. Human gamma-delta (γδ) T cells are a unique and conserved population of lymphocytes mediating innate immune surveillance as the first line of defense against infection and have been shown to be involved in potent antitumor responses [11]. According to their T cell receptor (TCR) delta chain usage, human γδ T cells are generally divided into two major subsets, namely Vδ1 and Vδ2 cells [12]. A small subset of Vδ3 T cells has been described, which plays a role in anti-cytomegalovirus (CMV) immunity [13]. γδ T cells account for 1–10% of all CD3 + circulating T cells but have been shown to expand in patients with hematological malignancies, such as lymphoma [14, 15], myeloma [16] and leukemia [17–20].

Pioneering studies by Lamb and colleagues have firmly established the important role of the graft-versus-leukemia effect (GvL) of γδ T cells in preventing the leukemia relapse in bone marrow transplantation [18, 21]. In an extended follow-up study, patients (*n* = 153) with high numbers of circulating γδ T cells showed significantly improved 5-year disease-free and overall survival after transplantation [22]. Significantly elevated γδ T cell counts were shown in peripheral blood and bone marrow in dasatinib-treated patients and on imatinib when compared to healthy controls [23]. Others found that among three TKIs, only dasatinib but not imatinib or nilotinib significantly enhanced the proliferation and antitumor responses of γδ T cells [24, 25]. Moreover, the expansions of monoclonal and oligoclonal T cells carrying clonal TCRγ and δ gene rearrangements have been observed in CML patients at diagnosis and during dasatinib therapy [26, 27]. The Vδ2 γδ T cells antileukemia reactivity has been shown in Ph + leukemia model [28] and more importantly in CML patients where Vδ2 T cells were shown to recognize and kill primary CML cells by TCR-mediated cytotoxicity [29]. Currently, no data are available on Vδ1 γδ T cell tumor reactivity in CML.

In the present study, we analyzed Vδ1 and Vδ2 T cells in CML patients at diagnosis and prospectively for the 18 months follow-up on TKI therapies. We determined the TCR clonality of Vδ1, Vδ2 and also Vγ9 chains in CML patients. Importantly, we show autologous cytotoxic reactivity of patients’ Vδ1 γδ T lymphocytes against primary CML cells.

## Materials and methods

### Patient characteristics

A total of 40 CML patients in chronic phase were enrolled at the Department of Internal Medicine, Hematology and Oncology, Faculty Hospital Brno in accordance with the Declaration of Helsinki and approved protocols by the Institutional review board and ethics committee of Masaryk University (#CCF09012015). All participants gave written informed consent. Patients were treated with imatinib (*n* = 22), dasatinib (*n* = 4), or nilotinib (*n* = 14). Clinical criteria including Sokal risk score were used. No patient selection was implemented. Peripheral blood (PB) samples were obtained at diagnosis and at 3, 6, 12 and 18 months following the TKI therapies. Patient characteristics are shown in Table 1.

**Table 1:** Patient characteristics

	CP-CML	EUROSKI	TKI (years) (median, range)	Off TKI (years) (median, range)
CML patients, *n*	40	9		
Age, years				
Median	62	65		
Range	21–81	46–83		
Sex, *n*				
Female	15	5		
Male	25	4		
TKI therapy, *n*				
Imatinib	22	6	6.5 (3.2–7.9)	2.45 (2.2–2.6)
Nilotinib	14	1	3.2	1.7
Dasatinib	4			
Imatinib/dasatinib		2	3.5/1.6; 0.7/4.3	1.2; 2.7
Sokal risk score				
Low	15			
Intermediate	17			
High	8			

*CP-CML* chronic phase CML, *TKI* tyrosine kinase therapy, *EURO-SKI* multicenter trial estimating the duration of major molecular remission (MMR) in CML patients after discontinuation the TKI

In addition, nine CML patients enrolled for the EURO-SKI multicenter trial estimating the duration of major molecular remission (MMR) in CML patients after discontinuation of the TKI were included. Patient characteristics including the TKIs, duration of the TKI therapy and the time off the TKI when the PB samples were analyzed are summarized in Table 1.

### Healthy donors

Peripheral blood samples from healthy volunteers and buffy coats (HD, *n* = 40) from age-matched donors (age 21–80 years, median 62 years) have been acquired at the Transfusion and Tissue Bank, Faculty Hospital Brno. All volunteers were in good and stable clinical conditions with no acute or chronic inflammatory diseases. Written informed consent was obtained.

### CML cell lines

Human chronic myeloid leukemia cell lines K562 were purchased from ATCC (American Type Culture Collection, USA), and LAMA-84 and KYO-1 and myeloma cell line U266 were purchased from DSMZ (German Collection of Microorganisms and Cell Cultures GmbH, Germany). Cells were cultured in RPMI-1640 containing 10% fetal bovine serum (FBS), 2 mM L-glutamine, 100 IU penicillin and 100 IU streptomycin (all Thermo Fisher Scientific).

### Separation of blood cells

Heparinized venous peripheral blood samples were processed by Lymphoprep (Stem Cell Technologies) within 2 h of collection using density gradient centrifugation. Isolated polymorphonuclear cells (PBMCs) were analyzed by flow cytometry or cryopreserved until use.

### Flow cytometry

Immunophenotyping was carried out including monoclonal antibodies (mAbs) against following antigens: CD3 (eBiosciences, clone SK7), CD27 (BD Pharmingen, clone M-T271), CD45RA (Exbio, clone MEM-56), Vδ1 (Thermo Fisher Scientific, clone TS8.2), Vδ1 (Miltenyi, clone REA173), Vδ2 (BD Pharmingen, clone B6). Freshly isolated PBMCs were labeled with antibodies and incubated at 4 °C for 30 min in the dark, washed twice in cold phosphate-buffered saline (PBS, Sigma) containing 2% fetal bovine serum (FBS) prior to analysis. Samples were acquired on a FACS CantoII (BD Biosciences) and analyzed using FACSDiva (BD Biosciences) and FlowJo software (Tree Star, Ashland, OR). Forward and side scatter gating was used to discriminate live cells from dead cells, and γδ T cells were derived from SSC vs FSC gated bulk PBMCs with doublet exclusion (FSC-A vs FCS-H). To determine the placement of the gates, appropriate fluorescence minus one (FMO) and unstained controls were used.

### Isolation of polyclonal Vδ1 and Vδ2 γδ T lymphocytes

Vδ1 and Vδ2 γδ T cell subsets were freshly sorted by positive selection using anti-TCR Vδ1 (Beckman Coulter, clone R9.12) or anti-TCR Vδ2 (BD Pharmingen, clone B6) monoclonal antibodies, magnetic microbeads and LS Columns (all Miltenyi Biotec, Germany) according to manufacturer’s instruction. The cell purity was routinely greater 97%.

### Cytotoxicity assay

Freshly sorted effector γδ T cells were co-cultured with CML target cells at indicated effector/target ratios (E:T) 5:1 and 10:1 in duplicates after 4 h co-culture at 37 °C as described previously [30]. Briefly, CML tumor cell lines or mononuclear cells (PBMC) isolated from CML patients at diagnosis were washed in Hank’s buffered saline solution (HBSS, Invitrogen Life Technologies) to remove FCS and culture media. Of note, the target CML tumor cells were not CD34 purified due to limited patient material. Cells were resuspended in diluent C (Sigma) and labeled with PKH67 fluorescent dye (Sigma). To-Pro-3 iodide (1 μM in PBS) (Invitrogen Life Technologies) was added immediately prior to the acquisition on the flow cytometer. At least 10,000 target cells were acquired after gating out the green fluorescence of PKH67 dye and the proportion of To-Pro-3 iodide-positive cells. Background target cell death was determined from the cells incubated in the absence of effector cells and was routinely below 10%.

### RNA extraction, cDNA synthesis, real-time PCR

Total RNA has been extracted from freshly isolated PBMC or sorted γδ T cells using RNeasy Mini kit (Qiagen) according to manufacturer’s instruction. RNA was eluted in RNAse-free water and stored in − 80 °C. Complementary DNA (cDNA) has been synthesized using 20 ng/µl total RNA that has been reverse transcribed using High Capacity cDNA Reverse Transcription Kit (Applied Biosystems). The glyceraldehyde 3-phosphate dehydrogenase (GAPDH) housekeeping gene has been used as an internal control by quantitative real-time polymerase chain reaction (real-time qPCR). cDNAs were amplified with specific primers for γ or δ chains using Premix TopTaq™ (Quiagen). Samples were analyzed by real-time PCR on StepOne™ Real-Time PCR Systems (Applied Biosystems). Primer sequences are listed in Supplementary Table SI.

### Detection of TCR γ and δ rearrangements by spectratyping analysis

Spectratyping analysis used labeled cDNA fragments separated by capillary electrophoresis on Genetic Analyzer Scan 3130 (Applied Biosystems). Results of intensity of fluorescence were analyzed by using GeneMapper® or Peak Scanner™ softwares. Clonality was determined visually on sample plots as follows: The TCR distribution was categorized as monoclonal (M)— one clone, biclonal (B)—two clones, oligoclonal (O)—3 to 5 clones and polyclonal (P)—6 and more clones.

#### Statistical analyses

Data analyses were performed using Prism® (GraphPad Software Inc., La Jolla, CA). Differences between sample groups were evaluated with the non-parametric Mann–Whitney *U*-test. *P* < 0.05 values were considered to be significant. Cytotoxicity results are expressed as mean ± standard deviation (SD). Median and range including min and max values are shown in relevant figures.

## Results

### γδ T cells in healthy donors and CML patients at diagnosis

**Fig. 1:**
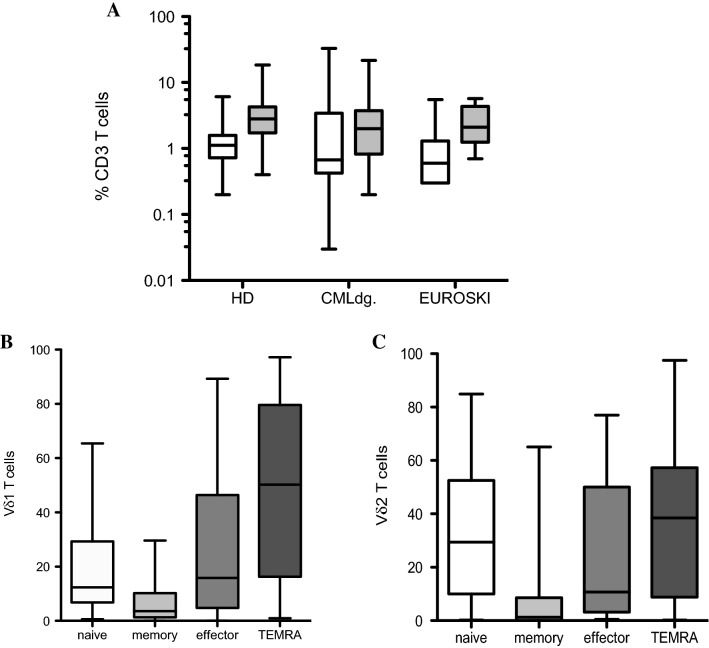
Flow cytometric analysis of Vδ1 and Vδ2 γδ T cells in newly diagnosed CML patients (*n* = 40) at diagnosis compared to age-matched healthy donors (HD, *n* = 40). Also, nine CML patients enrolled for the EURO-SKI after discontinuation the TKI were included. **A** Peripheral blood mononuclear cells (PBMCs) were analyzed for percentages of Vδ1 (white bars) and Vδ2 (gray bars) γδ T cells among leukocytes gate followed by the percentage of CD3 lymphocytes. Box and whiskers show min, max and median values. **B** Immunophenotyping of Vδ1 and Vδ2 (**C**) T cells using the CD27 and CD45RA antibodies to determine the naïve (CD27 + CD45RA +), memory (CD27 + CD45RA −), effector (CD27 − CD45RA −) and TEMRA (CD27 − CD45RA +) phenotypes was examined. Box and whiskers show min, max and median values

To determine the numbers and to characterize phenotypically the two major populations of γδ T lymphocytes, Vδ1 and Vδ2 T cell subsets from freshly isolated PBMCs of newly diagnosed CML patients in chronic phase (CP-CML, *n* = 40) were compared to frequencies of age-matched healthy donors (HD, *n* = 40). The EURO-SKI patients (*n* = 9) who discontinued the TKI were included in the analysis. No significant differences were found for Vδ1 T cells in CML patients at diagnosis (0.03–32.9%, median 0.7) compared to HD (0.2–6.1%, median 1.1). Similar results were detected for frequencies of Vδ2 T cells in HD (0.4–18.5%, median 2.8) and CML patients at diagnosis (0.2–21.7%, median 2.0) as shown in Fig. 1A. Additionally, Vδ1 T cells (0.3–5.5%, median 0.6) and Vδ2 T cells (0.7–5.7%, median 2.1) found in EURO-SKI patients were not significantly different to HD.

Phenotyping analyses of Vδ1 and Vδ2 T cells using CD27 and CD45RA antibodies to determine the naïve (CD27 + CD45RA +), memory (CD27 + CD45RA −), effector memory (CD27 − CD45RA −) and terminally differentiated (TEMRA, CD27 − CD45RA +) phenotypes showed the Vδ1 T cell subset being represented mostly in TEMRA phenotype as shown in Fig. 1B. The Vδ2 T cells were identified mostly as naïve and TEMRA phenotypes in Fig. 1C.

### Vδ1 and Vδ2 cells γδ T cells in CML patients during the TKI therapy

**Fig. 2:**
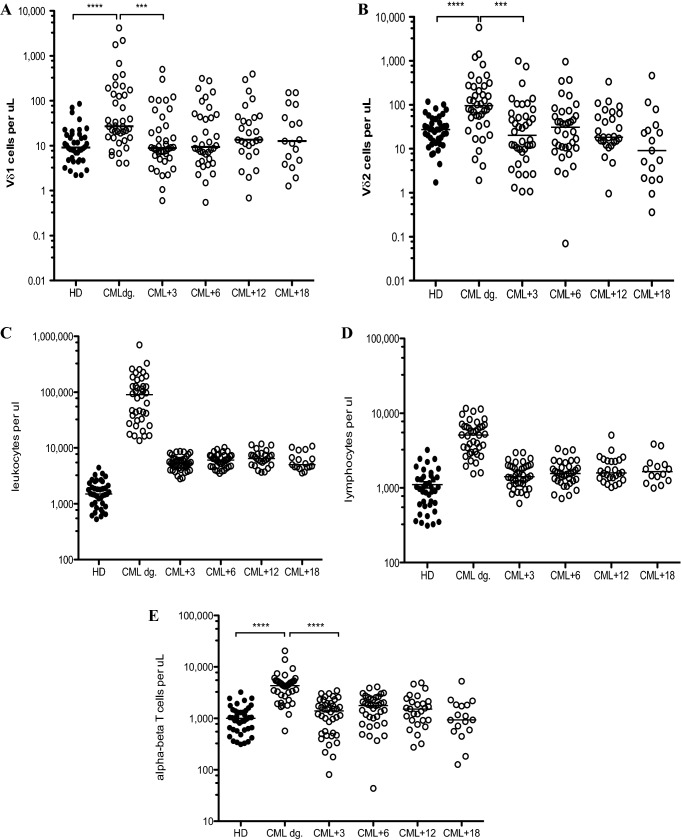
Summary of the Vδ1 and Vδ2 γδ T cell absolute counts in CML patients at diagnosis (CML dg., *n* = 40) and during the TKI therapy (open circles) compared to healthy donors (*n* = 40, black circles). PBMC samples were analyzed at diagnosis and at 3 months (CML + 3), 6 months (CML + 6), 12 months (CML + 12) and 18 months (CML + 18) for Vδ1 (A) and Vδ2 (B) γδ T cells. The median values are shown. Statistically significant differences are presented as **** *p* < 0.0001, *** *p* = 0.0001. Absolute leukocyte counts (C) and absolute lymphocyte counts (D) and absolute counts of αβ T cells (E) are shown with median values during the patient follow-up

First, we analyzed the absolute counts of Vδ1 and Vδ2 γδ T cells in patient peripheral blood samples at diagnosis and then at 3-, 6-, 12- and 18-month follow-ups and compared that to age-matched healthy donors. Interestingly, we found Vδ1 γδ T cells significantly expanded in newly diagnosed CML patients (*p* < 0.0001) (median 27.2 cells/µl; range 4.1- 4,166) compared to HD (median 9.1 cells/µl; range 2.2–85.6) as shown in Fig. 2A. Matching results were obtained for Vδ2 γδ T cells where we also observed significant expansions in CML patients at diagnosis (*p* < 0.0001) (median 96.2 cells/µl; range 1.9–5,789) compared to HD (median 27.4 cells/µl; range 1.7–118.2) as shown in Fig. 2B. These results reflect the leukocytosis (Fig. 2C) and also lymphocytosis (Fig. 2D) accustomed in CML patients at diagnosis. In addition, we have recounted the T cell pool and enumerated αβ T cells in each patient time-point as proportion of CD3positive/ Vδ1negative/Vδ2negative T cells. We show significantly increased absolute counts of αβ T cells in CML patients at diagnosis (*p* < 0.0001) (median 4,333 cells/µl; range 569.8–20,515) compared to HD (median 997 cells/µl; range 312–3,228) in Fig. 2E.

Absolute counts of both Vδ1 and Vδ2 γδ T cell subsets (*p* = 0.0001) and similarly absolute counts of αβ T cells dropped at 3 months (*p* < 0.0001), respectively, for each T cell populations into normal range of healthy donors correlating to the TKI therapies.

**Fig. 3:**
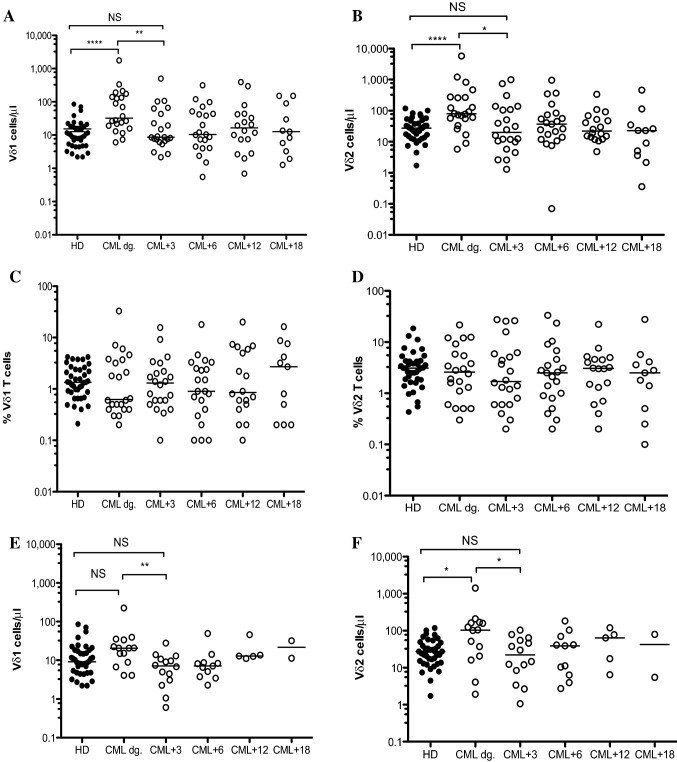

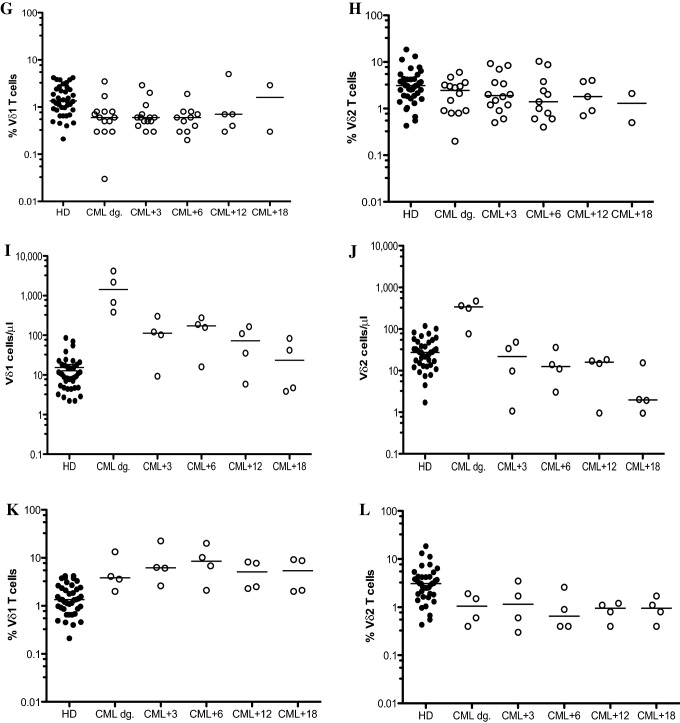
Reconstitution of Vδ1 and Vδ2 γδ T cells in CML patients during the TKI therapy. CML patients were divided at diagnosis into imatinib (*n* = 22), nilotinib (*n* = 14) and dasatinib (*n* = 4) cohorts. Absolute numbers of Vδ1 (**A**) and Vδ2 (**B**) and frequencies of Vδ1 (**C**) and Vδ2 (**D**) γδ T cells in patients on imatinib compared to HD (*n* = 40) were determined at diagnosis (CML dg.) during the 18 months therapy as 3 months (CML + 3), 6 months (CML + 6), 12 months (CML + 12) and 18 months (CML + 18). Similarly, Vδ1 (**E**) and Vδ2 (**F**) γδ T cells and frequencies of Vδ1 (**G**) and Vδ2 (**H**) γδ T cells in patients on nilotinib were analyzed. Finally, absolute counts of Vδ1 (**I**) and Vδ2 (**J**) γδ T cells and frequencies of Vδ1 (**K**) and Vδ2 (**L**) γδ T cells on dasatinib (*n* = 4) are presented. The median values are shown. Statistically significant differences are presented as **** *p* < 0.0001, ** *p* < 0.001 and * *p* < 0.01

Based on the results above, we divided CML patients who had initiated the TKIs into cohorts including imatinib (*n* = 22), nilotinib (*n* = 14) and dasatinib (*n* = 4). The γδ T cell reconstitution in CML patients on imatinib showed significant reductions of both Vδ1 T cell (*p* = 0.003) and Vδ2 T cell (*p* = 0.02) absolute counts at 3 months compared to that of diagnosis as shown in Fig. 3A and B. However, Vδ1 and Vδ2 T cell frequencies in CML patients on imatinib were not significantly different during the 18-month TKI therapy as shown in Fig. 3C and D. Similarly, CML patients on nilotinib therapy significantly decreased the Vδ1 T cells (*p* = 0.008) and Vδ2 T cells (*p* = 0.02) absolute counts at 3 months follow-up compared to diagnosis (Fig. 3E, F); frequencies of Vδ1 and Vδ2 T cells in nilotinib cohort showed no significant differences during the 18-month TKI follow-up as shown in Fig. 3G and H. The γδ T cell reconstitution in absolute counts (Fig. 3I, J) and frequencies (Fig. 3 K, L) in four patients on dasatinib is shown.

In addition, we have also enumerated αβ T cells in patient cohorts including imatinib (*n* = 22), nilotinib (*n* = 14) and dasatinib (*n* = 4) during the 18 months therapy as shown in Supplemental Fig. 1. We showed significant decrease of αβ T cells in CML patients at 3 months follow-up compared to that of diagnosis for all three TKIs.

In summary, these results showed both major γδ T cell subsets significantly expanded in CML patients at diagnosis and subsequently significant reductions of absolute counts reflecting the TKI therapies lowering the Vδ1 and Vδ2 γδ T cells to normal levels.

### Reconstitution of γδ T cell subsets based on Sokal score in CML patients at diagnosis and at 3 months post-TKI therapy

**Fig. 4:**
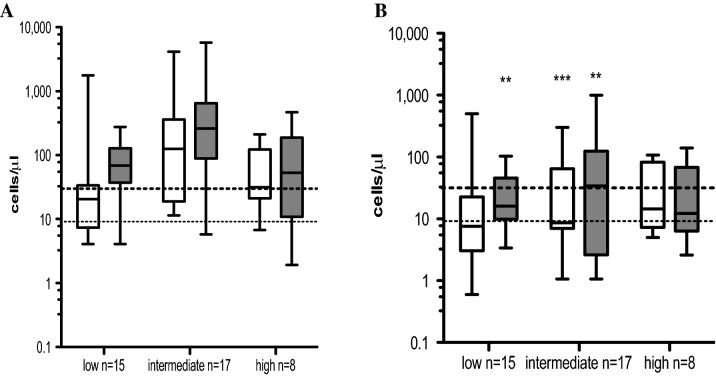
Summary of absolute numbers of Vδ1 and Vδ2 γδ T cell subsets based on Sokal risk score at diagnosis and at 3 months post-TKI therapy. CML patient cohort was divided in 3 × groups based on Sokal score into low (*n* = 15), intermediate (*n* = 17) and high (*n* = 8). Absolute numbers of Vδ1 (white bars) and Vδ2 (gray bars) γδ T cells are shown at diagnosis (**A**) and at 3 months follow-up (**B**). Box and whiskers show min, max and median values. Dotted line represents the median values of Vδ1 T cell counts, and dashed line represents the median values of Vδ2 T cells from healthy donors (*n* = 40). Statistically significant differences are presented as ** *p* = 0.002, *** *p* = 0.0007

Next, we stratified CML patients based on Sokal risk score at diagnosis into low (*n* = 15), intermediate (*n* = 17) and high (*n* = 8) groups. Patients in all groups showed both Vδ1 and Vδ2 γδ T cell subsets represented above the normal ranges at CML diagnosis (Fig. 4A). However, patients in the low Sokal risk score group (*n* = 9 on imatinib, *n* = 6 on nilotinib), intermediate Sokal risk score group (*n* = 8 on imatinib, *n* = 5 on nilotinib, *n* = 4 on dasatinib) and high Sokal risk score group (*n* = 5 on imatinib, n = 8 on nilotinib) presented Vδ1 γδ T cells at 3 months post-TKI significantly reduced in the intermediate Sokal risk score cohort (*p* = 0.0007) (Fig. 4B). The Vδ2 T cell subset showed decreased counts at 3 months post-TKI in the low Sokal risk score group (*p* = 0.002), in the intermediate Sokal risk score group (*p* = 0.008) as shown in Fig. 4B.

**Fig. 5:**
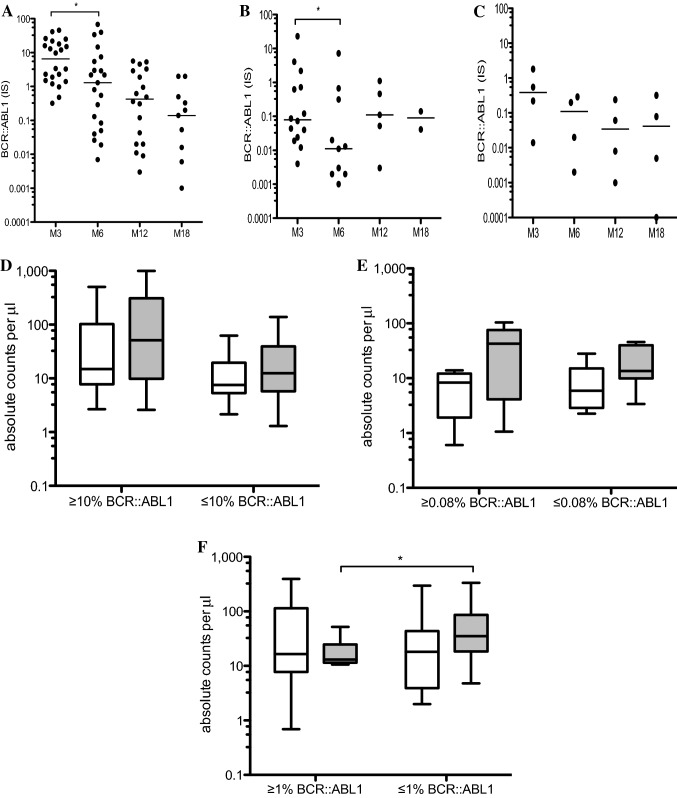
Molecular response as the % BCR::ABL1 (IS) in CML patients. (**A**) Patients on imatinib (*n* = 22), (**B**) patients on nilotinib (*n* = 14) and (**C**) patients on dasatinib (*n* = 4) were analyzed and median values are shown during the 18 months TKI therapies defined at 3 months (M3), 6 months (M6), 12 months (M12) and 18 months (M18). Statistically significant differences are presented as * *p* = 0.02. The median values are shown. (**D**) summary of Vδ1 and (white bars) Vδ2 (gray bars) γδ T cells in patients on imatinib at 3 months with BCR::ABL1 (IS) > 10% (*n* = 11) and optimal responders (*n* = 11) with BCR::ABL1 (IS) > 10% vs BCR::ABL1 (IS) ≤ 10%. (**E**) Vδ1 (white bars) and Vδ2 (gray bars) γδ T cells in patients on nilotinib at 3 months with 0.08% BCR::ABL1 (IS) > 0.08% (*n* = 8) and optimal responders with BCR::ABL1 (IS) ≤ 0.08% (*n* = 6). (**F**) Vδ1 (white bars) and Vδ2 (gray bars) γδ T cells in patients on imatinib at 12 months with BCR::ABL1 (IS) > 1% (*n* = 6) and optimal responders with BCR::ABL1 (IS) ≤ 1% (*n* = 12). Box and whiskers show min, max and median values. Statistically significant differences are presented as ** *p* = 0.04

Additionally, we examined the molecular response, namely the % BCR::ABL1 (IS) in CML patients and throughout the 18-month follow-up, and found significant reductions of BCR::ABL1 from 3 months compared to 6 months in patients on imatinib (*p* = 0.02) and its further gradual decrease during the TKI therapy as shown in Fig. 5A. Similarly, significant reductions of BCR::ABL1 in patients on nilotinib (*p* = 0.03) were found as shown in Fig. 5B. The four patients on dasatinib are shown in Fig. 5C.

Early molecular response has been shown to have strong prognostic value for each of the TKIs used in the frontline therapy. BCR::ABL1 transcripts (IS) < 10% at 3–6 months separate patients into high and low risk categories for progression and long-term outcome [31]. Therefore, we compared Vδ1 and Vδ2 γδ T cells in patients on imatinib (*n* = 22) at 3 months with BCR::ABL1 (IS) > 10% vs BCR::ABL1 (IS) ≤ 10%. Patients with BCR::ABL1 (IS) > 10% (*n* = 11) showed higher but not significantly Vδ1 and also Vδ2 γδ T cell counts than cohort of optimal responders (*n* = 11) with BCR::ABL1 (IS) ≤ 10% (Fig. 5D). Similarly, we analyzed Vδ1 and Vδ2 γδ T cells in patients on nilotinib at 3 months (*n* = 14). We used the median value of 0.08% BCR::ABL1 (IS) determined in our patient cohort. Vδ1 T cells showed no difference in patients with BCR::ABL1 (IS) > 0.08% (*n* = 8) and BCR::ABL1 (IS) ≤ 0.08% (*n* = 6). However, Vδ2 γδ T cells were detected at higher levels in patients BCR::ABL1 (IS) > 0.08% compared to optimal responders with BCR::ABL1 (IS) ≤ 0.08% (Fig. 5E). Together, these results suggest that higher absolute counts of Vδ1 and Vδ2 T cells found in patients with higher levels of tumor burden and BCR::ABL1 reflects the ongoing expansion of γδ T lymphocytes mediating antileukemic responses. Patients on dasatinib (*n* = 4) were not analyzed due to small numbers. In several studies, the achievement of a complete cytogenetic response (CCyR) as BCR::ABL1 transcripts (IS) ≤ 1% at 12 months or later on TKI therapy was associated with a significant survival benefit compared with achievement of lesser degrees of response [31]. We analyzed γδ T cells in patients on imatinib (*n* = 18) at 12 months with BCR::ABL1 (IS) > 1% vs BCR::ABL1 (IS) ≤ 1%. We found more Vδ1 T cells in optimal responders with BCR::ABL1 (IS) ≤ 1% (*n* = 12) when compared to patients with BCR::ABL1 (IS) > 1% (*n* = 6). Importantly, significantly more Vδ2 T cells (*p* = 0.04) were also identified in BCR::ABL1 (IS) ≤ 1% CML patient group (Fig. 5F). Patients on nilotinib (*n* = 5) and dasatinib (*n* = 4) at 12 months were not analyzed due to small numbers.

### Vδ1 γδ T cell-mediated killing of CML tumor targets

**Fig. 6:**
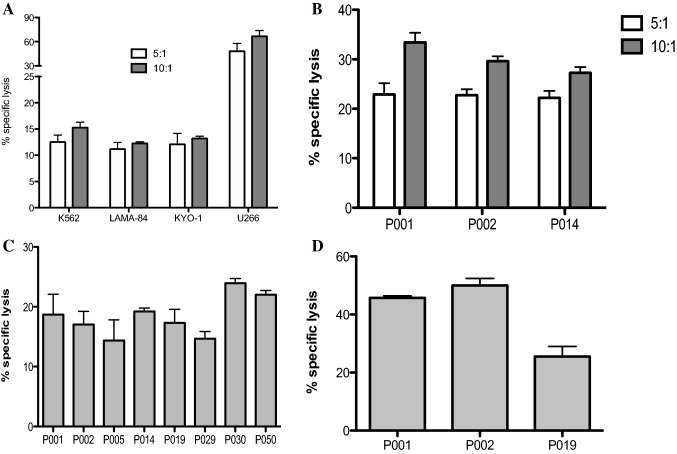
Vδ1 T cell-mediated killing of CML targets. Freshly sorted Vδ1 γδ T cells from healthy donors were co-cultured with CML targets for 4 h, and specific lysis was determined at 5:1 (white bars) and 10:1 (gray bars) effector/ target (E:T) ratio. (**A**) Summary of the results from independent experiments from five healthy donors against CML cell lines including K562 (CML blast crisis), LAMA-84 and KYO-1 and against multiple myeloma U266 cell line is shown, with the mean ± SD of sample triplicates. (**B**) Cytotoxic reactivity data of Vδ1 γδ T cells isolated from healthy donors against primary CML cells isolated from PBMCs from patients (P001, P002 and P014) at diagnosis. Data shown are mean ± SD of independent experiments performed in duplicates. (**C**) Summary of inherent cytotoxicity from eight CML patients’ autologous Vδ1 γδ T cells against primary CML cells. Patient numbers are shown. Data shown are mean ± SD of independent experiments performed in duplicates. (**D**) Specific lysis of CML patients’ Vδ1 γδ T cells against allogeneic primary CML cells. Patient numbers are shown. Data shown are mean ± SD of independent experiments performed in duplicates

We aimed to determine the cytotoxic reactivity of γδ T cells shown as percentages of specific lysis of CML targets. Since the Dieli’s group clearly showed that Vδ2 γδ T cells efficiently recognize and kill Zoledronate-treated CML cells [29], we focused primarily on freshly sorted Vδ1 γδ T cells. First, highly pure Vδ1 T cells isolated from healthy donors were tested for their cytotoxic function against BCR-ABL^+^ CML cell lines including K562 (blast crisis), LAMA-84 and KYO-1 shown in Fig. 6A. All of the tested γδ T lymphocytes isolated from five HD showed low reactivity to CML tumor cell lines at 5:1 and 10:1 E:T ratio. Percentage of specific lysis at 5:1 ranged between K652 (mean 15.3%, SD 1.1%), LAMA-84 (mean 12.3%, SD 0.3%), KYO-1 (mean 13.2%, SD 0.4%). However, the same effector Vδ1 γδ T cells killed efficiently the multiple myeloma U266 tumor targets (mean 64.2%, SD 8.8%) at the same 5:1 E:T ratio. Similar results showing prominent killing function of Vδ1 γδ T cells from healthy donors against myeloma and lymphoma cell lines have been published in our previous study [30]. Next, we determined the cytotoxic reactivity Vδ1 T cells isolated from healthy donors (*n* = 3) against primary CML cells isolated from CML patients at diagnosis shown in Fig. 6B. We observed higher percentage of specific lysis at 5:1 ratio (mean 22.6%, SD 0.3%) and at 10:1 (mean 30.1%, SD 3.0%) compared to CML cell lines. Furthermore, killing of primary CML tumor cells has been detected in autologous patients’ Vδ1 γδ T cells (Fig. 6C). All of the eight tested patient Vδ1 T cell samples were able to lyse CML tumor at low 5:1 ratio (mean 18.4%, SD 3.3%). Also, we determined the cytotoxic reactivity of CML patients’ Vδ1 γδ T cells against allogeneic primary CML cells at 5:1 ratio (mean 40.4%, SD 7.5%) shown in Fig. 6D. Together, we demonstrate the inherent ability of Vδ1 T cells to kill CML primary tumor cells.

### Spectratyping analysis of γδ TCR in healthy donors and CML patients during the TKI therapies

**Fig. 7:**
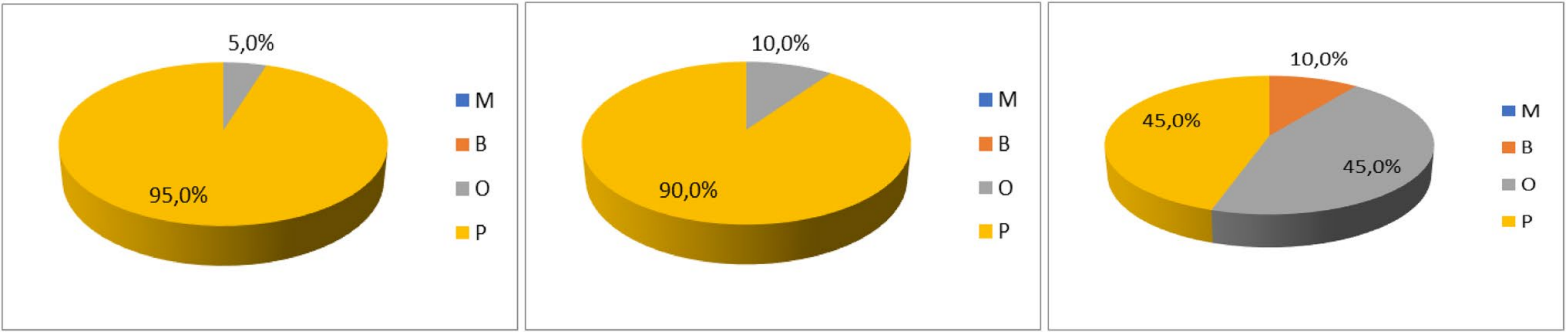
CDR3 clonality of γδ T cell subsets in healthy donors. The TCR repertoire was determined for the Vδ1, Vδ2, and Vγδ9 chains in age-matched healthy donors (*n* = 20). Pie charts visualize of the clonality in % (**A**) Vδ1chain, (**B**) Vδ2 chain, (**C**) Vγ9 chain as M (monoclonal, 1 clone, in blue), B (biclonal, 2 clones, in orange), O (oligoclonal, 3–5 clones, in gray), P (polyclonal, 6 and more clones, in yellow)

To address the γδ T cell clonality of the Vδ1, Vδ2, and Vγ9 chains, we performed the TCR repertoire analysis previously published [13]. First, clonality of Vδ1, Vδ2, and Vγ9 chains was analyzed in age-matched healthy donors (*n* = 20) and the results are summarized in Fig. 7A-C and in Supplemental Table SII. Overall majority of the Vδ1 and Vδ2 chains were detected as polyclonal. The Vγ9 chain clonality showed equal samples polyclonal and oligoclonal (9/20) and two biclonal samples.

Second, we determined the TCR repertoire in CML patients at diagnosis and during the TKI therapies including imatinib (*n* = 22), dasatinib (*n* = 4), nilotinib (*n* = 14). Summary of the results is shown in Table 2 (Vδ1), in Table 3 (Vδ2), in Supplementary file 3, Vγ9). We show that Vδ1 and Vδ2 T cells in CML patients on dasatinib were predominantly polyclonal. Similarly, majority of imatinib- and nilotinib-treated patients showed Vδ1 and Vδ2 T cells polyclonal profiles. Of interest, the Vγ9 chain repertoires were identified mostly oligoclonal regardless of the TKI.

**Table 2:**
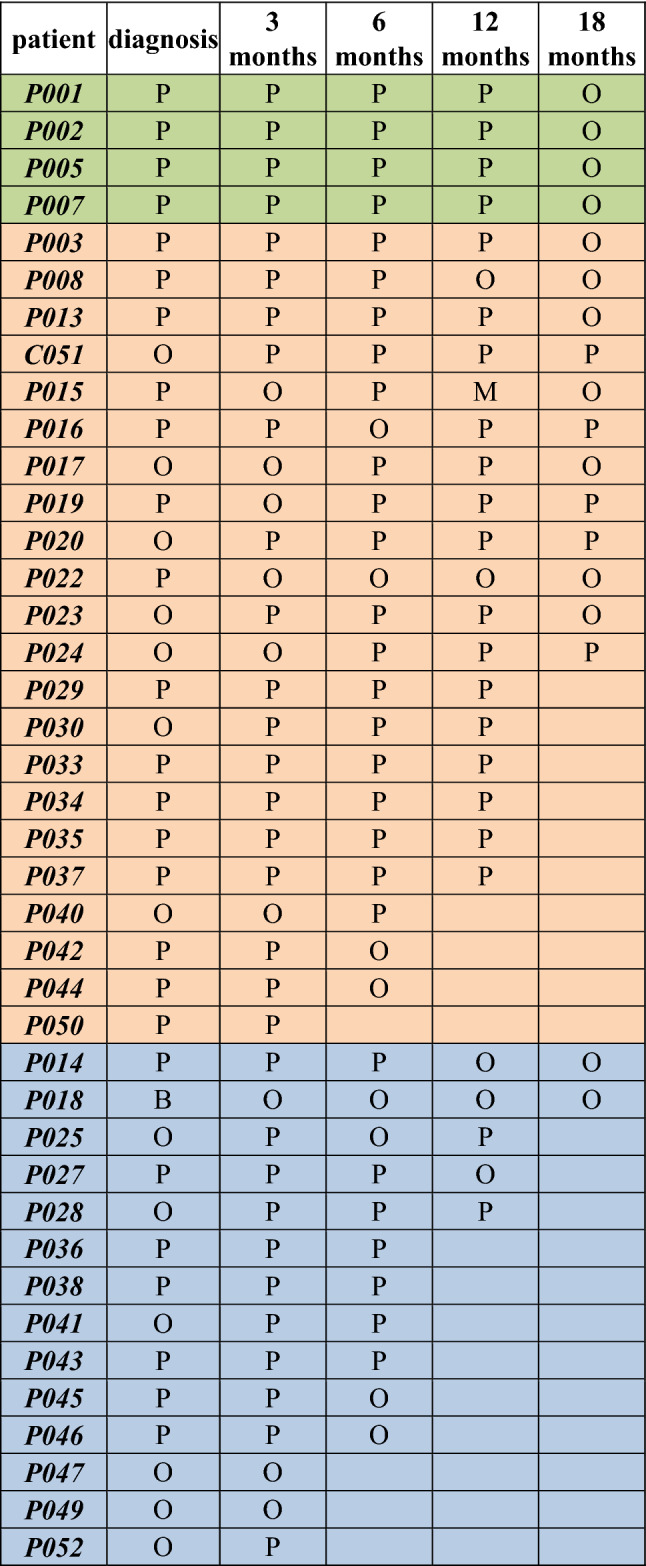
Summary of the TCR clonal distribution of the Vδ1 chain in CML patients**.** The TCR repertoire in CML patients at diagnosis and during the TKI therapies including imatinib (*n* = 22, in orange), dasatinib (*n* = 4, in green), nilotinib (*n* = 14, in blue) is shown as M (monoclonal, 1 clone), B (biclonal, 2 clones), O (oligoclonal, 3–5 clones), P (polyclonal, 6 and more clones)

**Table 3:**
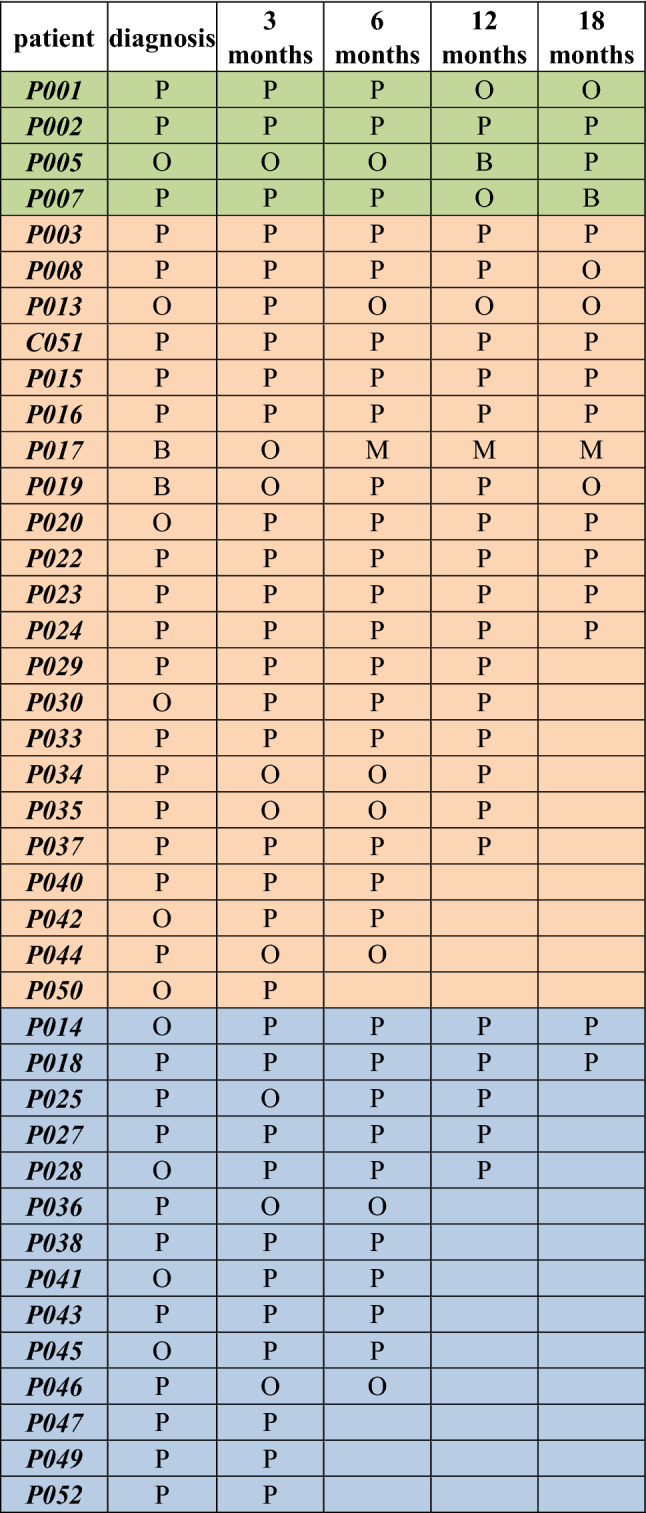
Summary of the TCR clonal distribution of the Vδ2 chain in CML patients**.** The TCR repertoire in CML patients at diagnosis and during the TKI therapies including imatinib (*n* = 22, in orange), dasatinib (*n* = 4, in green), nilotinib (*n* = 14, in blue) is shown as M (monoclonal, 1 clone), B (biclonal, 2 clones), O (oligoclonal, 3–5 clones), P (polyclonal, 6 and more clones)

## Discussion

γδ T cells are one of the key players of the innate effector immunity substantially contributing to tumor elimination and have been identified as the most significant favorable prognostic immune subset associated with overall survival outcomes across 39 malignancies in a large analysis of transcriptomic expression signatures from ~ 18,000 human tumors [28]. Specifically, tumor-infiltrating Vγ9Vδ2 γδ T cell gene signatures were re-analyzed and were identified to correlate with favorable prognosis in ~ 10,000 cancer biopsies from 50 types of malignancies with prominent abundance in B-cell acute lymphoblastic leukemia, acute promyelocytic leukemia (M3-AML) and as well as CML [29].

In the present study, we showed for the first time, distributions of both major populations of Vδ1 and Vδ2 γδ T cells in newly diagnosed CP-CML patients and prospectively throughout the first 18 months TKI therapies including imatinib, dasatinib and nilotinib. At diagnosis, CML patients showed significantly expanded γδ T cell numbers of both Vδ1 and Vδ2 γδ T cells when compared to age-matched healthy donors. Immunophenotyping of Vδ1 T cell subset at diagnosis showed mostly the TEMRA (CD27-CD45RA +), while the Vδ2 T cells were identified as naïve (CD27 + CD45RA +) and TEMRA phenotypes suggesting ongoing stimulation of the effector γδ T cell immunity. Recent study has shown an increased frequency of Tregs and CD8 + effector cells displaying phenotype of T cell exhaustion in CML at diagnosis and in patients with refractory disease [32]. Importantly, expression of immune checkpoint molecules such as TIM-3 and LAG-3 at diagnosis was reduced in patients after achieving molecular response (MR3) following dasatinib treatment but increased again in patients at hematological relapse [32]. It is apparent that T cells represented by conventional αβ CD8 + effector cells and also by γδ T cells in CML patients at diagnosis display alterations in a) numbers, where significant expansions in both major γδ T cell subsets were shown in our study and in particular in b) phenotype of exhaustion/terminal differentiation.

To examine the effect of individual TKI on the reconstitution of γδ T cell subsets, we examined CML patients divided into imatinib, dasatinib and nilotinib cohorts and found that dasatinib-treated patients presented significantly expanded γδ T cells in agreement with previous report [17]. The γδ T cell reconstitution in imatinib and nilotinib CML groups showed significant reductions of both Vδ1 and Vδ2 T cell counts at 3 months post-TKI to normal levels where they remained throughout the 18 months follow-up. Importantly, when we assessed early molecular response in patients in high- and low-risk categories based on BCR::ABL1 transcripts (IS) < 10% versus BCR::ABL1 (IS) > 10% at 3 months [31], we found in imatinib-treated patients higher Vδ1 and Vδ2 γδ T cell counts in a cohort of non-optimal responders. Similarly, more Vδ1 and Vδ2 γδ T cells were found in non-optimal responders on nilotinib at 3 months post-TKI. These results may argue for the ongoing expansion of innate effector γδ T lymphocytes mediating anti-leukemic responses at early TKI therapy. Additionally, we found higher levels of Vδ1 and Vδ2 T cells in optimal responders at 12 months in imatinib cohort, which may suggest the γδ T cells ‘ contribution to improved disease outcome.

The EURO-SKI patients, who discontinued the TKI therapy, presented both Vδ1 and Vδ2 γδ T subsets at normal levels. Additionally, we showed that the increased absolute counts of αβ T cells in CML patients at diagnosis then dropped at 3 months (*p* < 0.0001) in response to imatinib and nilotinib TKI therapies similarly to both γδ T cell subsets.

Several studies have highlighted the γδ T cells cytotoxic function against primary tumor cells in myeloid leukemias. The analysis of Vγ9Vδ2 T cells in newly diagnosed acute myeloid leukemia (AML) showed efficient killing of autologous AML blasts following activation by the phosphorylated bromohydrin (BrHPP) analog that mimics the biological properties of natural phospho-antigen [33]. The Dieli’s group clearly showed that Vγ9Vδ2 γδ T cells efficiently recognized and killed Zoledronate-treated CML cells [29]. Both studies, however, have not analyzed Vδ1 T cells, and currently, no data are available on Vδ1 γδ T cell antitumor reactivity in CML. Therefore, we determined the cytotoxic reactivity of Vδ1 γδ T cells freshly separated from healthy donors against primary CML tumor cells isolated from PBMC from CML patients at diagnosis. We showed percentages of specific lysis by Vδ1 γδ T cells at low 5:1 and 10:1 effector/target ratio without any prior stimulation or priming by antigens. Tumor killing by Vδ1 T cells against primary CML was comparable to that of stimulated Vγ9Vδ2 γδ T cells against CML cells [26] or AML blasts [34]. Importantly, we described autologous Vδ1 γδ T cell-mediated killing of primary CML cells. These findings provide new evidence for the inherent antileukemia reactivity of Vδ1 T cells in CML patients.

Next, we determined the TCR repertoire for the Vδ1, Vδ2, and Vγ9 chains in CML patients at diagnosis to established the impact of each TKI on the γδ T cell reconstitution. Previous studies have shown the expansions of monoclonal and oligoclonal T cells carrying clonal TCRγ and δ gene rearrangements in CML patients at diagnosis and during dasatinib therapy (22, 23). In contrast, in our study, we found that Vδ1 and Vδ2 T cells in CML patients on dasatinib were predominantly polyclonal. Similarly, majority of imatinib- and nilotinib-treated patients showed Vδ1 and Vδ2 T cells polyclonal profiles suggesting unrestricted potential of anti-CML immune responses in long-term disease control regardless of the TKI.

The use of TKIs has dramatically transformed the treatment of CML patients. Studies have explored the potential of the TKI discontinuation in selected patients who maintain a deep molecular response for 2 years on TKI treatment [35–37]. Over the past decade, the TKI therapy discontinuation has shown promising results for a number of CML patients in clinical trials and is increasingly explored as a novel immunomodulatory approach to treat the disease. The EURO-SKI clinical trial enrolled CML patients at 61 European centers in 11 countries showed that the majority of patients who discontinue therapy after 3 years on TKI would remain in remission at least for 1 year; ~ half of the patients experienced molecular relapse within the first 6 months after treatment discontinuation [38].

More clinical trials together with biological comparison studies are still needed to determine clinical success of the TKI therapies and involvement of γδ T cell populations including their anti-leukemia effector mechanisms and how these cells exert their therapeutic effects during the TKI cessation as it was shown for NK cells during treatment-free remission [39, 40]. In this regard, enhancement of the potent of γδ T cell function is a promising strategy for ongoing research to design new γδ cell-based immunotherapies [41–44].

## Supplementary Information

Below is the link to the electronic supplementary material.

Supplementary file1 (DOCX 27 KB)

Supplementary file2 (PPTX 140 KB)

Supplementary file3 (DOCX 15 KB)

## Data Availability

Data are available upon request. All data relevant to the study are included in the article.
