# Supplementary material for: Expansions of tumor-reactive Vdelta1 gamma-delta T cells in newly diagnosed patients with chronic myeloid leukemia
Source: Cancer Immunol Immunother. 2022 Nov 14;72(5):1209–24. doi: 10.1007/s00262-022-03312-3 (PMC10110709; doi:10.1007/s00262-022-03312-3)
Supplement: Supplementary file 1 — Supplementary file1 (DOCX 27 KB) [file 262_2022_3312_MOESM1_ESM.docx]

**Supplementary material**

Primer sequences for the CDR3 analysis are listed in Supplementary Table SI.

| **Primer** | **Sequence** | **Company** |
| --- | --- | --- |
| V δ1 | 5‘-CTG TCA ACT TCA AGA AAG CAG CGA AAT C-3‘ | Eurofins Genomics |
| V δ2 | 5‘-TAC CGA GAA AAG GAC ATC TAT GGC-3‘ | Eurofins Genomics |
| V δ3 | 5‘-GGG GAT AAC AGC AGA TCA GAA GGT-3‘ | Eurofins Genomics |
| C δ | 5‘-TGG GAG AGA TGC AAT AGC AGG ATC-3‘ | Eurofins Genomics |
| V γ9 | 5‘-CAG CCC GCC TGG AAT GTG TGG-3‘ | Sigma |
| J γ | 5‘-CAA CAA GTG TTG TTC CAC TGC C-3‘ | Eurofins Genomics |
| **Primer** | **Sequence** | **Company** |
| C δ FAM | 5‘-ACG GAT GGT TTG GTA TGA GGC TGA-3‘ | Eurofin Genomics |
| Jγ1.3 FAM | 5‘-[6FAM]CAA CAA GTG TTG TTC CAC TGC C-3‘ | Sigma |

**Table SII:** Summary of the TCR clonal distribution in healthy donors**.** M (monoclonal, 1 clone), B (biclonal, 2 clones), O (oligoclonal, 3-5 clones), P (polyclonal, 6 and more clones).

| HD | Vδ1 | Vδ2 | Vγ9 |
| --- | --- | --- | --- |
| 1 | P | P | P |
| 2 | P | P | P |
| 3 | P | P | P |
| 4 | P | P | O |
| 5 | P | P | O |
| 6 | P | P | P |
| 7 | P | P | P |
| 8 | P | P | O |
| 9 | P | O | P |
| 10 | P | P | B |
| 11 | P | P | P |
| 12 | P | P | O |
| 13 | P | P | O |
| 14 | P | P | O |
| 15 | P | O | P |
| 16 | P | P | P |
| 17 | P | P | B |
| 18 | P | P | O |
| 19 | O | P | O |
| 20 | P | P | O |
