# Supplementary figures and images for: Expansions of tumor-reactive Vdelta1 gamma-delta T cells in newly diagnosed patients with chronic myeloid leukemia
Source: Cancer Immunol Immunother. 2022 Nov 14;72(5):1209–24. doi: 10.1007/s00262-022-03312-3 (PMC10110709; doi:10.1007/s00262-022-03312-3)

## Slide 1
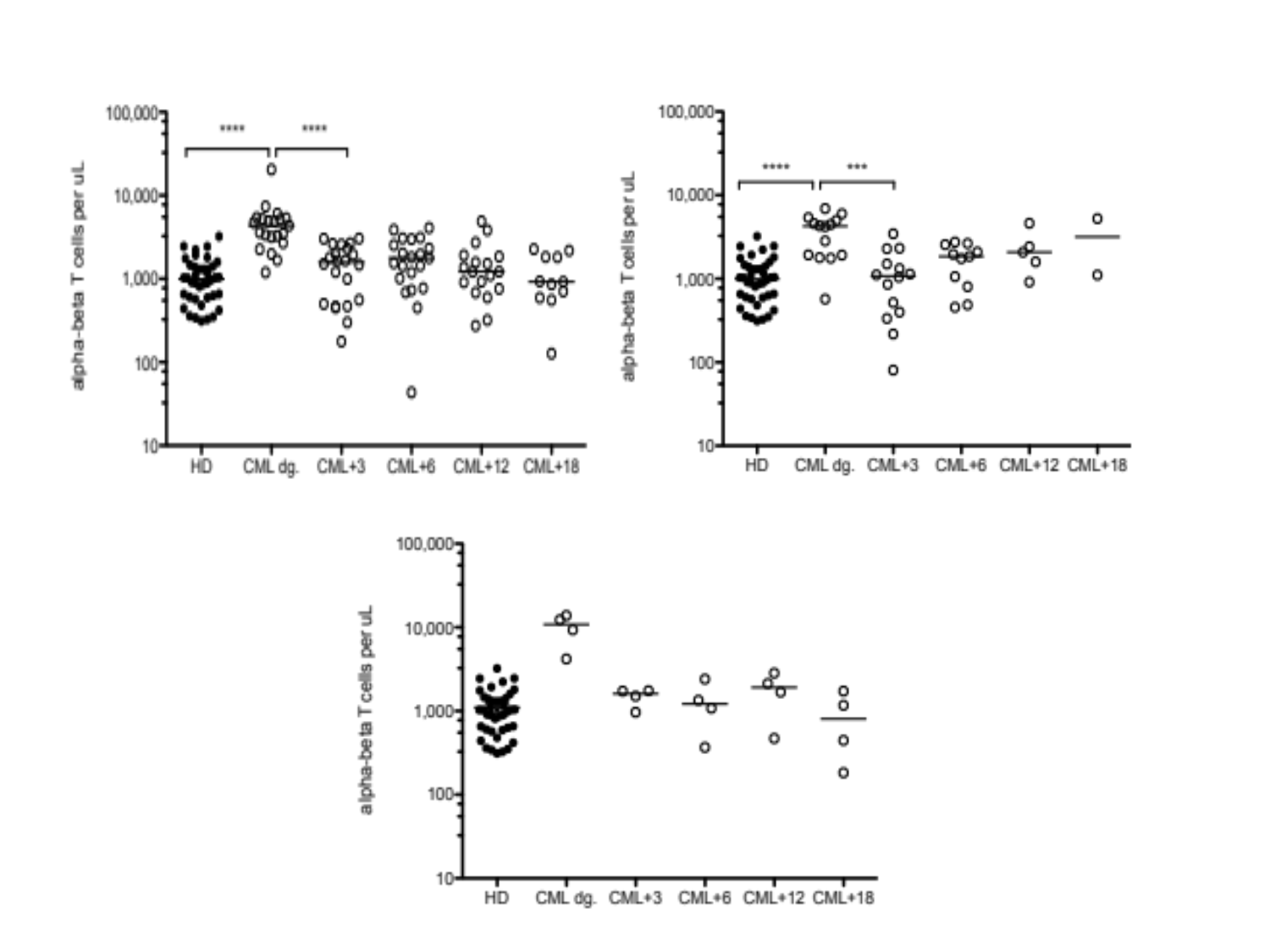

Supplement: Supplementary file 2 — Supplementary file2 (PPTX 140 KB) [file 262_2022_3312_MOESM2_ESM.pptx]
