# Supplementary material for: Expansions of tumor-reactive Vdelta1 gamma-delta T cells in newly diagnosed patients with chronic myeloid leukemia
Source: Cancer Immunol Immunother. 2022 Nov 14;72(5):1209–24. doi: 10.1007/s00262-022-03312-3 (PMC10110709; doi:10.1007/s00262-022-03312-3)
Supplement: Supplementary file 3 — Supplementary file3 (DOCX 15 KB) [file 262_2022_3312_MOESM3_ESM.docx]

**Table 4:** Summary of the TCR clonal distribution of the Vγ9 chain in CML patients**.** The TCR repertoire in CML patients at diagnosis and during the TKI therapies including imatinib (n = 22, in orange), dasatinib (n = 4, in green), nilotinib (n = 14, in blue) is shown as M (monoclonal, 1 clone), B (biclonal, 2 clones), O (oligoclonal, 3-5 clones), P (polyclonal, 6 and more clones).

| **patient** | **diagnosis** | **3 months** | **6 months** | **12 months** | **18 months** |
| --- | --- | --- | --- | --- | --- |
| ***P001*** | M | O | O | M | O |
| ***P002*** | B | B | B | B | B |
| ***P005*** | M | B | O | B | O |
| ***P007*** | O | O | O | O | B |
| ***P003*** | P | P | P | O | B |
| ***P008*** | O | O | O | O | O |
| ***P013*** | M | O | O | M | O |
| ***C051*** | P | P | P | O | O |
| ***P015*** | O | O | O | P | P |
| ***P016*** | M | B | M | M | M |
| ***P017*** | B | O | M | M | M |
| ***P019*** | O | O | O | O | O |
| ***P020*** | O | O | O | O | O |
| ***P022*** | O | B | O | P | P |
| ***P023*** | O | O | O | O | O |
| ***P024*** | M | M | M | M | O |
| ***P029*** | O | O | B | P |  |
| ***P030*** | O | M | O | B |  |
| ***P033*** | O | O | O | P |  |
| ***P034*** | O | O | B | O |  |
| ***P035*** | O | O | O | O |  |
| ***P037*** | P | O | O | M |  |
| ***P040*** | B | O | P |  |  |
| ***P042*** | O | P | P |  |  |
| ***P044*** | B | B | O |  |  |
| ***P051*** | O | O |  |  |  |
| ***P014*** | O | B | O | O | O |
| ***P018*** | O | B | O | P | P |
| ***P025*** | M | O | O | O | B |
| ***P027*** | O | B | O | O |  |
| ***P028*** | O | B | P | P |  |
| ***P036*** | P | O | O | O |  |
| ***P038*** | O | O | O |  |  |
| ***P041*** | O | M | P |  |  |
| ***P043*** | P | P | M |  |  |
| ***P045*** | O | O | P |  |  |
| ***P046*** | B | B | P |  |  |
| ***P047*** | P | P |  |  |  |
| ***P049*** | O | O |  |  |  |
| ***P052*** | P | P |  |  |  |
